# Migration of Apicomplexa Across Biological Barriers: The *Toxoplasma* and *Plasmodium* Rides

**DOI:** 10.1111/j.1600-0854.2008.00703.x

**Published:** 2008-02-20

**Authors:** Isabelle Tardieux, Robert Ménard

**Affiliations:** 1 Institut Cochin, Université Paris Descartes, Centre National de la Recherche Scientifique UMR 8104 75014 Paris, France; 2 Institut National de la Santé et de la Recherche Médicale U567 Paris, France; 3 Unité de Biologie et Génétique du Paludisme, Institut Pasteur 28 Rue du Dr Roux, 75015 Paris, France

**Keywords:** Apicomplexa zoites, host cell invasion, host cell traversal, *Plasmodium*, *Toxoplasma*

## Abstract

The invasive stages of Apicomplexa parasites, called zoites, have been largely studied in *in vitro* systems, with a special emphasis on their unique gliding and host cell invasive capacities. In contrast, the means by which these parasites reach their destination in their hosts are still poorly understood. We summarize here our current understanding of the cellular basis of *in vivo* parasitism by two well-studied Apicomplexa zoites, the *Toxoplasma* tachyzoite and the *Plasmodium* sporozoite. Despite being close relatives, these two zoites use different strategies to reach their goal and establish infection.

Apicomplexa constitute a large phylum of parasitic protozoa. Many are pathogenic to humans like *Plasmodium*, the causative agent of malaria, and *Toxoplasma*, which induces severe manifestations in immunocompromised individuals, while others like *Eimeria* and *Theileria* cause heavy losses in domestic animals and cattle. They are obligate intracellular parasites that invade host cells by developing into specialized stages called zoites. Zoites have a conserved structure, being elongated and polarized cells that secrete at their anterior tip the content of apically located secretory organelles, named micronemes and rhoptries (1). Zoites move on solid substrates by gliding, without changing their overall shape (2), at the impressive speed of several microns per second. This motility, which is powered by a linear actomyosin motor located underneath the zoite plasma membrane (3,4), also allows zoite invasion of host cells inside a so-called parasitophorous vacuole (PV), a process that lasts only a few seconds.

Zoite entry inside a PV is typically followed by parasite development and the generation of multiple new zoites. Some but not all Apicomplexa zoites express a second, more dramatic way to invade host cells by piercing their plasma membrane and migrating through them (5). Two of the three zoites of *Plasmodium*, the ookinete and the sporozoite, traverse host cells, like sporozoites of *Toxoplasma* and *Eimeria*. The *Toxoplasma* tachyzoite, however, does not traverse host cells.

So far, most studies on Apicomplexa zoites have been performed *in vitro*, focusing in particular on the *Toxoplasma* tachyzoite and the *Plasmodium* sporozoite. These studies have greatly boosted our molecular understanding of gliding motility and of parasite interactions with cultured cells. In contrast, we know little of how these zoites behave in their hosts. We summarize below recent findings on the ways by which the *Toxoplasma* tachyzoite and the *Plasmodium* sporozoite traffic inside host tissues and across cellular barriers to disseminate and establish infection.

## The *Toxoplasma* Tachyzoite

**Figure 1:**
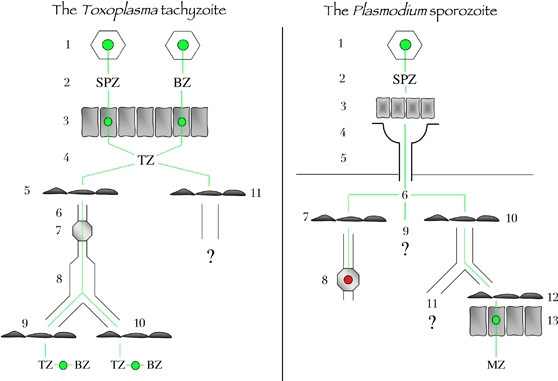
Schematics of *in vivo* infection by the *Toxoplasma* tachyzoite and the *Plasmodium* sporozoite Left, the *Toxoplasma* tachyzoite. *Toxoplasma* infection starts by the ingestion of oocysts or tissues cysts (1) that liberate in the intestinal lumen free sporozoites or bradyzoites, respectively (2). Free parasites invade enterocytes (3), where they multiply and transform into tachyzoites, which are released in the lamina propria of the intestine (4). Tachyzoites invade the endothelium of lymphatic vessels (5), are taken up by the lymph (6), go through lymph nodes (7) and reach the blood circulation (8). Tachyzoites then cross blood endothelium barriers in immunoprivileged organs (9), for example, the brain, the retina or the placenta, or in other organs (10), for example, muscles, where they invade host cells, multiply and transform into dormant bradyzoites (11). Tachyzoites in the intestine may also cross the endothelium of blood vessels. Right, the *Plasmodium* sporozoite. Sporozoites are formed inside oocysts (1) in the wall of the mosquito midgut, are released in the hemocele bathed by the hemolymph (2), invade acinar cells (3) and exit in the secretory cavities (4) of the salivary glands and finally move into the secretory ducts (5). In the mammalian host, sporozoites are deposited in the dermis (6). Sporozoites can invade the endothelium of lymphatic vessels in the dermis (7) and then end up in the proximal draining lymph node (8), where they are killed. Sporozoites can remain in the dermis (9), in which case their fate is unknown. They can invade the endothelium of blood vessels in the dermis (10), reach the liver, cross the endothelium barrier of liver sinusoids (12) and invade hepatocytes (13), where they transform into merozoites, the erythrocyte-infecting form of the parasite. Sporozoites in the blood can also reach other organs than the liver (11), for example, the spleen, where the sporozoite fate is not well documented. BZ, bradyzoite; MZ, merozoite; SPZ, sporozoite; TZ, tachyzoite.

*Toxoplasma gondii* is a water-/food-borne parasite that can subvert any warm-blooded animal as host and cause severe pathology (6). Following oral ingestion, the parasite initially crosses the intestinal epithelium and disseminates in numerous tissues including immunoprivileged sites such as the central nervous system, the retina or a developing fetus. Infection is initiated either by the sporozoite form, after ingestion of water/food contaminated by oocysts from cat feces, or by the bradyzoite form, after ingestion of cyst-containing raw meat. Both the sporozoite and the bradyzoite forms are released from their enclosing structures in the intestinal lumen, penetrate intestinal epithelial cells and subsequently differentiate into the rapidly replicating tachyzoite stage (Figure 1). The tachyzoites must then disseminate from the intestinal lamina propria, where they are released, to the distant sites where they can further replicate inside cells and eventually differentiate into the bradyzoite form, a quiescent parasite stage as long as the immune system holds it in check within a cyst.

The success of the tachyzoite essentially depends on its ability to reach and cross endothelia from both lymphatic and blood vessels. The tachyzoite dissemination is thought to start by the infection of gut-associated secondary lymphoid organs (7), and recent quantitative polymerase chain reaction analysis supports the view that tachyzoites traffic through the intestinal lymphatic vessels before reaching the blood (8). At the other end of the blood transport, tachyzoites cross blood endothelial barriers into tissues, particularly the placenta in primo-parasitized pregnant females and the blood–brain and blood–retina barriers, which lead to the most severe pathology. Since free tachyzoites are known to survive several hours in serum-containing medium and to invade virtually any nucleated host cell type, the central question is whether they reach their destination by using their own motility or by hijacking host circulating leukocytes.

### Traveling as an extracellular parasite

Evidence that tachyzoites can travel extracellularly to their destination by an active process is mainly indirect. Type I (RH) *Toxoplasma* strains, known to rapidly disseminate and generate high tissue burdens in murine models, are associated with a stronger migratory capacity, that is, a greater proportion of gliding individuals moving longer distances, compared with the less virulent type II and type III strains (9). Type I tachyzoites better disseminate *ex vivo* in mouse ileum explants, with more parasites entering the lamina propria and penetrating the submucosa and in some cases reaching the vascular endothelium (9,10). They also more efficiently cross Madin Darby Canine Kidney (MDCK)-polarized cells cultured as monolayers on a transwell (11). In this system, free tachyzoites migrate across monolayers by passing between cells, that is, using a paracellular route. They gather mainly around intercellular junctions and actively migrate across the cellular barrier without altering its integrity. Migration between MDCK cells was also proposed to depend on interactions between intercellular adhesion molecule 1 (ICAM-1) on host cell surfaces and the parasite protein micronemal protein 2 (MIC2) (11), which links the membrane-associated motor to the extracellular ligands during parasite gliding (12). In contrast, tachyzoites did not appear to use a transcellular route to cross the MDCK barrier, that is, they did not enter cells apically and exit from the basolateral side.

Although it remains to be seen whether tachyzoites interact with endothelial barriers, particularly from the brain, in the same manner as they interact with MDCK epithelial barriers, these data favor the view that tachyzoites might disseminate to distant tissues as extracellular parasites by an active process. During parasite transmigration, the interactions between MIC2, which contains an I-domain, and ICAM-1 are reminiscent of the interactions between I-domains of β2 leukocyte integrins and immunoglobulin folds present in many intercellular junctional molecules during leukocyte diapedesis (13). However, although such interactions typically mediate leukocyte passage by a paracellular route, they also allow a transcellular route of diapedesis by which leukocytes induce the formation of and migrate through intracellular ‘transmigratory cups’ across individual endothelial cells (14).

### Hijacking motile leukocytes

More recent work suggests that the tachyzoite might also subvert host cells to reach its destination. Tachyzoites are known to actively penetrate various types of leukocytes *in vitro*(15,16) and to localize inside leukocytes in the murine intestine (8). It was shown recently (8) that following intragastric delivery of parasite cysts in mice (i) tachyzoites parasitize CD11c+ dendritic cells in the lamina propria of the intestine and the mesenteric lymph nodes, suggesting that these cells contribute to the early parasite dissemination from the intestinal wall; (ii) in the blood, parasites associate with CD11b+, most likely monocytes (rather than B cells or neutrophils), but not CD11c− leukocytes; (iii) the CD11c+ dendritic cells and the CD11b+ leukocytes recovered from the mesenteric lymph nodes and the blood of parasitized mice, respectively, can trigger the parasitic process once intravenously transferred to naïve mice and in both cases reach the brain and (iv) anti-CD11b blocking antibodies prevent CD11b+ circulating leukocytes from extravazating into tissues. This indicates that the parasitized blood CD11b+ monocytes can migrate across the blood–brain barrier and promote parasite entry in the brain.

**Figure 2:**
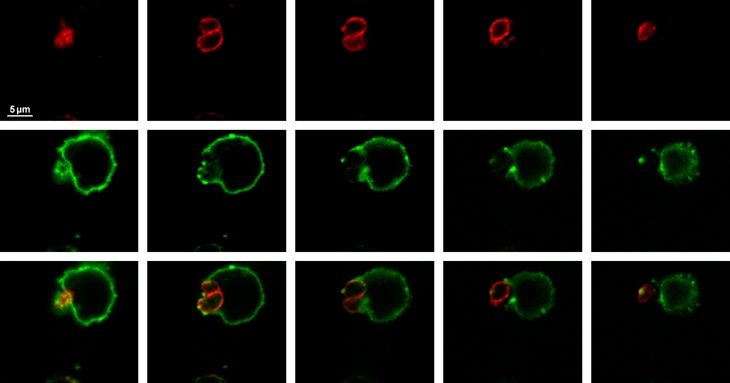
A pair of tachyzoites is associated with MHC-II positive cells in lymph nodes A mouse was infected with cysts of the 76K *Toxoplasma gondii* strain, mesenteric lymph nodes were recovered 5 days after infection, a cell suspension from the lymph node was fixed, MHC class II molecules were detected using class II I-A^bdq^ and I-E^dk^ antibodies (green), the parasite surface was stained using anti-SAG1 antibodies (red) and samples were observed by confocal microscopy (0.4-μm section). Note that the tachyzoites are located at the periphery of the host cell and are surrounded by the plasma membrane of the host cell. MHC, major histocompatibility complex.

Intriguingly, only single or paired tachyzoites were found to be associated with the dendritic cells from the mesenteric lymph nodes and the blood CD11b+ cells, while typical intracellular rosettes indicative of dividing tachyzoites were rarely seen associated with the shuttle cells (8). In most cases, the single parasite seemed intracellular but localized at the cell periphery and did not appear to be located inside a typical PV. Rather, the intracellular but growth-arrested parasite was wrapped within folds of the host cell plasma membrane, in what could be a novel type of interaction between the *Toxoplasma* tachyzoite and the host cells (Figure 2). This transport interaction could ensure that the parasite is shielded but does not start replicating inside the shuttle cell and thus avoids its lysis and death.

There is also evidence that the hijackers alter the motile properties of the shuttle leukocytes. *In vivo*, leukocyte extravazation is sixfold greater in parasitized mice than in nonparasitized mice (8). Bioluminescence *in vivo* imaging also showed that mice inoculated with tachyzoite-loaded dendritic cells suffered wide parasite dissemination and developed dramatically higher parasite loads, including in the brain, earlier than mice inoculated with free parasites (17,18). *In vitro*, bone marrow-derived dendritic cells containing tachyzoites exhibit significantly enhanced transmigration across endothelial cell monolayers in transwells, while uninfected dendritic cells and tachyzoite-loaded monocytes or fibroblasts do not. This induced hypermotility phenotype depends on the presence of live, not phagocytosed parasites and is associated with an upregulation of maturation markers and costimulatory molecules. Manipulation of dendritic cells by the tachyzoites seems specific because dendritic cells harboring tachyzoites do not upregulate ICAM-1, unlike lipopolysaccharide-matured dendritic cells, and slightly downregulate the CD11a and CD18 integrin chains (17).

Therefore, these data support the concept that transport of tachyzoites in migratory leukocytes contributes significantly to their dissemination *in vivo*, in particular to the brain. However, it remains unclear whether the CD11c+ cells characterized *in vitro*(17) play a role in dissemination *in vivo* because following parasite inoculation through the natural route (8), infected CD11c+ cells were only found in the secondary lymphoid organs and not in the blood. In addition, dendritic cells infected *in vitro*(17) contained rosettes, whereas the dendritic cells *in vivo* harbored only single or paired parasites (8). However, the exact nature of the interaction between the tachyzoite and its shuttle cell, that is, surrounded by plasma membrane extensions or truly internalized, awaits better characterization. Direct visualization, possibly by two-photon microscopy, of blood–brain barrier crossing events should be decisive in determining the leukocyte subpopulation that effectively shuttles tachyzoites to the brain.

## The *Plasmodium* Sporozoite

The life of the *Plasmodium* sporozoite is a perilous odyssey from its site of birth, the midgut wall of an Anopheline mosquito, to its destination, a hepatocyte in a mammalian host (Figure 1). There, the sporozoite finally settles to generate tens of thousands of merozoites, the parasite form that is adapted to erythrocytes and initiates the pathogenic replication cycles. To reach the hepatocyte, the sporozoite relies on two basic abilities: a vigorous gliding motility and an aggressive cell transmigration behavior.

### Marathon man

Much direct evidence has accumulated in the past few years indicating that the *Plasmodium* sporozoite remains extracellular during most of its journey and locomotes through its own active motility and passive transport in the fluids of its hosts. In the mosquito, although sporozoites need not be motile for leaving the oocyst and reaching the hemolymph, active motility is needed for penetrating the secretory cells of the salivary glands and reaching the extracellular secretory cavities (19). Sporozoites also move by gliding inside salivary cavities and ducts and apparently need to access the ducts to be ejected during salivation (20). During the mosquito bite, most sporozoites are inoculated into the dermis of the mammal (21,22), as mosquitoes inject saliva while probing the skin, before ingesting blood. Imaging *Plasmodium berghei* sporozoites in the mouse ear has shown the strong, tortuous and apparently random motility of the few individuals immediately after inoculation (23,24). The duration of sporozoite active motility appears to be greater *in vivo* than *in vitro* and might also vary with the parasite species; while *P. berghei* sporozoites are no longer motile after 2 h (24), *Plasmodium yoelii* sporozoites trickle out from the skin into the blood for hours after the mosquito bite (25,26). Sporozoites also glide beyond the dermis, including along endothelial surfaces inside dermal blood vessels (27), inside the lymph node (24) and in the sinusoids and the liver parenchyma (28,29).

The picture of the *Plasmodium* pre-erythrocytic phase that emerges from intravital imaging studies is more complex than the traditional view of all injected sporozoites traveling from the skin to the liver through the blood (Figure 1). Instead, the skin phase of the sporozoite's life appears to act as a crossroad, the dermal sporozoites having three possible fates; (i) they can invade blood capillaries in the dermis (23,24) and reach the liver, where they invade hepatocytes; (ii) they can invade lymphatic vessels in the dermis (24), in which case they do not end up in the liver, as had been hypothesized (30,31), but stop their journey in the proximal lymph node, where most parasites are eventually degraded inside dendritic cells (24) and (iii) they can be left in the dermis after cessation of their active motility, and the fate of the sporozoites left in the dermis remains to be characterized (24,32). Although this picture results from studies on rodent-infecting *Plasmodium* species, the original observation by Boyd and Kitchen (21) of a *Plasmodium vivax* sporozoite in a draining lymph node 24 h after sporozoite injection by a mosquito suggests that human- and rodent-infecting sporozoites might behave similarly in their respective host.

### Crossing endothelial barriers in the skin: which way in?

**Figure 3:**
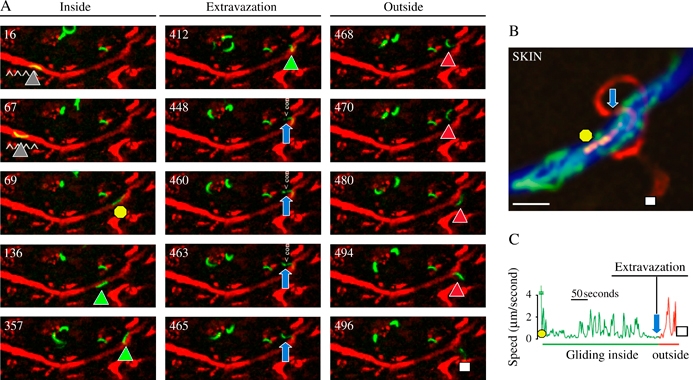
A *Plasmodium berghei* sporozoite exiting a blood vessel in the dermis of a mouse A) The fluorescent sporozoite glides inside the vessel colored in red after injection of red fluorescent BSA. The time (in seconds) is indicated in the upper left part of each panel. The intravascular sporozoite glides during the first 67 seconds (gray triangles), is suddenly displaced (yellow circle, 69 seconds), glides again inside the vessel (green triangles), extravazates (from 448 to 465 seconds, note the sporozoite constriction pointed by the blue arrows) before gliding in the dermis (468 to 496 seconds, red triangles until the white square). B) Maximum intensity projection of the fluorescent sporozoite from the 69th (yellow circle) to the 496th second (white square) through the constriction (blue arrow). C) Velocity profile of the sporozoite between the 69th and the 496th second.

Little is known of how sporozoites cross endothelial barriers. Intravital imaging shows that sporozoites are able to cross endothelial blood barriers in the dermis in both directions, in (24) and out (Figure 3) of the vessel lumen. To do this, sporozoites could use their cell traversal capacity, which will be examined in more detail below. However, the mutants lacking the proteins sporozoite protein essential for cell traversal (SPECT) or SPECT2 known to be specifically defective in host cell traversal (33,34) can still cross both blood and lymphatic endothelia in the skin (35). Cell traversal might still be involved in crossing endothelia but depend on membrane-damaging molecules other than SPECT/SPECT2. One such possibility is a phospholipase possessing a domain homologous to mammalian lecithin–cholesterol acyl transferases, which is important for the sporozoite capacity to leave the skin after natural transmission (36), although the exact defect of the phospholipase null mutant, that is, lack of cell traversal or otherwise, remains unknown. Alternatively, by analogy with the *Toxoplasma* tachyzoite, sporozoites might pull on junctional molecules to transmigrate by a paracellular route or, like leukocytes, through transcellular channels without breaching cell plasma membranes.

### Leaving the blood in the liver: which way out?

**Figure 4:**
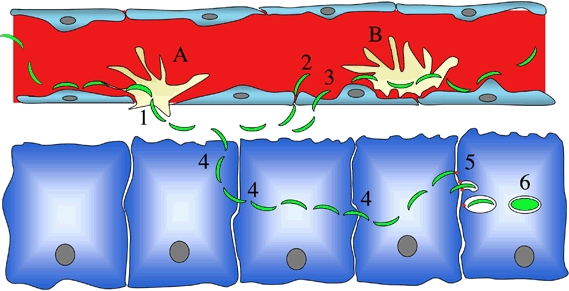
How does the *Plasmodium* sporozoite leave the lumen of the liver sinusoid and invade a hepatocyte inside a PV? It is still unclear whether Kupffer cells are embedded in the sinusoidal barrier (A) or sit on the top of endothelial cells (B). If (A) occurs, then traversal of Kupffer cells (either by transcytosis, i.e., involving sporozoite entry inside a PV followed by escape from the cell, or by disrupting the Kupffer cell membranes and migrating through the cell) would be sufficient for crossing the sinusoidal barrier. If (B) occurs, then the sporozoite may, after traversing Kupffer cells, cross the endothelial barrier either by a paracellular route (2) or by traversing endothelial cells (3). Once in the parenchyma, the sporozoite traverses several hepatocytes (4) before invading a final one inside a PV (5), the only niche where a sporozoite can fully develop (6). In two opposing views, the final invasion step is either activated by prior hepatocyte traversal or instead constitutively available and requires inhibition of cell traversal.

Crossing the liver sinusoidal barrier to reach hepatocytes poses a specific problem (Figure 4). The liver sinusoids are lined by endothelial cells and harbor resident macrophages, the Kupffer cells, which phagocytose particulate and foreign materials from the portal circulation. The role of these phagocytic cells during sporozoite passage into the liver parenchyma has been a much debated question, ever since the first transmission electron microscopy (TEM) evidence that Kupffer cells might act as gates by bridging the sinusoidal lumen and underlying hepatocytes (37). An unresolved issue is the exact position of Kupffer cells in the sinusoid wall. Kupffer cells are known to be less motile than other leukocytes in the sinusoids, or even immotile, but whether they are interspersed between endothelial cells or instead lie on their luminal face is still unclear.

The work from one laboratory has provided evidence supporting the ‘gateway hypothesis’(28,^38^–41), in line with the view that Kupffer cells dwell between endothelial cells, and proposed that Kupffer cell traversal is an obligatory step of the parasite life cycle (42). Still, the final demonstration by intravital imaging that sporozoites translocate from the sinusoid lumen into the parenchyma through Kupffer cells (28) has been difficult to provide, as acknowledged by the authors themselves (41), because of the insufficient resolution of the wide-field microscopy used. One traditional argument against the gateway hypothesis is the fact that clodronate, which kills Kupffer cells and other macrophages, greatly enhances sporozoite infection of the liver (43). However, the authors have suggested using TEM that the clodronate-induced macrophage death leaves temporary gaps in the sinusoidal barrier large enough to be used by sporozoites as artificial gates, although too small to cause hemorrhage into the parenchyma (42). Genetically altered mice (*op*/*op*) having fewer Kupffer cells because of a defect in macrophage maturation were also shown to be more resistant to sporozoite infection (42), although the pleiotropy of the *op* mutation, causing for example the liver and hepatocytes to be much smaller in mutant mice, makes specific conclusions on sporozoite translocation into the parenchyma uncertain.

An alternative hypothesis would place most Kupffer cells on the top of endothelial cells (44–46) and would assume that traversal of Kupffer cells does not obviate the need to cross the endothelial barrier. Kupffer cells, like dermal macrophages (see below), would essentially play a detrimental role on sporozoite progression, providing a simple explanation to the effect of clodronate. Traversal of Kupffer cells would be followed by translocation across the sinusoidal barrier, which could occur by one of the mechanisms mentioned above for sporozoites on the way in. In any case, the use of clodronate clearly demonstrates that Kupffer cells are not important for sporozoite infection, regardless of how sporozoites cross the barrier in their absence.

## Making Sense of Host Cell Traversal: Activating Cell Infection in the Liver Or Escaping Phagocytosis *En Route* to the Liver?

In addition to being able to infect hepatocytes, that is, penetrating them inside a PV, the *Plasmodium* sporozoite can also traverse host cells, that is, glide through them. The sporozoite cell traversal capacity was first described by Vanderberg et al. (47) when imaging interactions between *P. berghei* sporozoites and rodent peritoneal macrophages. Among other types of interactions, sporozoites were seen entering and exiting macrophages in a ‘needling manner’ and inducing an ‘outward flow of host cell cytoplasm at the point of egress’. Host cell traversal was also shown to occur with epithelial cells and fibroblasts (48). The role that this cell traversal behavior plays in sporozoite infection *in vivo*, however, remains controversial.

Because *in vivo* cell traversal by sporozoites was documented first in the liver parenchyma of rodent hosts (28,48), it was presumed that cell traversal would somewhat favor hepatocyte infection (48). In fact, it was reported that traversing several hepatocytes was essential to render sporozoites competent for infecting a ‘final hepatocyte’ inside a vacuole by regulated exocytosis of thrombosponding related anonymous protein (TRAP) (the *Toxoplasma* MIC2 ortholog) and other micronemal products important for the moving junction (MJ) and PV biogenesis (49). This seemed counterintuitive, though, as TRAP-dependent gliding motility by definition precedes any cell traversal event, and sporozoites build a MJ and a PV to penetrate salivary gland cells in the mosquito (50). Subsequent work suggested that cell traversal also had an impact on the host hepatocyte (51) in that migration through hepatocytes induced the secretion of hepatocyte growth factor from wounded cells, which in turn activated MET-dependent signals in neighboring infected cells. These signals were first proposed to be essential for parasite differentiation by reorganizing actin around the PV (51) and later to act mainly by preventing apoptosis in the infected cell (52). A model thus emerged in which hepatocyte traversal would enable the two subsequent steps of the parasite life cycle: hepatocyte infection, by activating the sporozoite, and parasite development, by priming the hepatocyte (53–56).

These conclusions, however, were questioned by the discovery of two proteins involved in sporozoite cell traversal named SPECT and SPECT2 (33,34), the latter containing a typical membrane-attack/perforin-like domain found in pore-forming proteins. Inactivation in *P. berghei* of either *spect*(33) or *spect2*(34) abrogates the sporozoite capacity to traverse but not to infect or develop inside, hepatocytes, thus arguing against both aspects of the above model. Intravital imaging recently showed that sporozoite mutants are immobilized and destroyed by phagocytic leukocytes in the dermis (35), in line with previous work providing indirect evidence for a role of cell traversal in resistance to Kupffer cells (33,34), thus supporting the view that cell traversal is primarily a means of defense against host phagocytic leukocytes. Once the sporozoite has penetrated the liver parenchyma, however, the traversal activity seems dispensable, and even harmful, to hepatocyte infection. Indeed, infection of primary hepatocytes is constitutive (completed in a few minutes) in the absence of cell traversal but is retarded in its presence (35). Therefore, the phenotype of the cell traversal-deficient mutants suggests the model that the cell traversal activity must be ‘on’ during the sporozoite journey to hepatocytes but should be switched off upon arrival to destination, hepatocyte infection resulting from repression of the traversal activity rather than from activation of the infection capacity.

Interestingly, a recent study proposes that the sporozoite uses the sulfation level of heparan sulfate proteoglycans (HSPG) on the surface of host cells as a Global Positioning System (57), helping it to decide whether to continue to migrate (through cells expressing under-sulfated HSPG) or to switch to invasion inside a vacuole (into cells covered with highly sulfated HSPG, primarily hepatocytes). This signal might thus promote sporozoite invasion upon cell contact, but whether it is also involved in arresting cell traversal remains unknown. Another recent study (58) shows that incubation of sporozoites in potassium almost abolishes cell traversal, suggesting that traversing cells might reduce the traversal activity itself. More work is needed for understanding what triggers the formation of the MJ and PV and the inhibition of cell traversal as well as the fine-tuning of these processes.

## Conclusions

It is clear that the *Toxoplasma* tachyzoite and the *Plasmodium* sporozoite, typically presented as exchangeable models, share common mechanisms to glide in host tissues and invade host cells inside a vacuole and possibly also common means of crossing cellular barriers. However, one key distinctive feature between the two zoites is the ability or inability to traverse host cells, which determines the different ways in which they interact with leukocytes and control their fate. During their life, both zoites rapidly encounter hostile phagocytes in the intestinal lamina propria for the newly formed tachyzoite and in the dermis for the freshly inoculated sporozoite. While the tachyzoite invades and hijacks leukocytes to locomote in the host, the sporozoite glides through them to find its way to the appropriate niche. The sporozoite also exhibits a vigorous gliding phenotype to go along with its brute-force strategy.

There are many questions left unanswered on how the *Toxoplasma* tachyzoite and the *Plasmodium* sporozoite reach their final niche. There is accumulating evidence that shuttle leukocytes play a major role in the dissemination of *Toxoplasma* tachyzoites, and investigating the mechanisms by which they subvert and pilot the host cell to destination promises to yield fascinating insights. However, the infectious potential of free tachyzoites is still unclear. They might target specific sites, including the placenta, and indeed, specific destinations in the host might be determined by the engagement of distinct host–parasite interactions. The journey of the *Plasmodium* sporozoite is also riddled with uncertainties. The impact on the host immune system of those sporozoites that do not reach a hepatocyte (59), most crucially in human infections, is a pressing question. How sporozoites cross endothelia, whether Kupffer cells act as gates or sieves in the process and how they switch from a traversal to an infective mode are still open questions. Because Apicomplexa zoites have multiple ways to interact with and cross cellular barriers and end up in different tissues in their hosts, the next challenge will be to recognize the relative contributions of each possible route of infection in order to fully grasp the versatility of these parasites and the complexity of the infections they cause. In that endeavor, *in vivo* imaging approaches will be crucial by examining host–parasite interactions in a natural context and in a quantitative manner.
